# Book Notices

**Published:** 1879-07-20

**Authors:** 


### EDITORIAL AND MISCELLANEOUS.

### *BOOK NOTICES.*

*AMERICAN HEALTH PRIMERS*, now being issued by Lindsay & Blakiston, are as follows. Price, 50 cents each The first two volumes are now ready, and others will follow at intervals of about one month: I. Hearing, and How to Keep It, by Chas. H. Burnett, M.D., of Philadelphia, Consulting Aurist to the Pennsylvania Institution for the Deaf and Dumb, Aurist to the Presbyterian Hospital, etc. II. Long Life, and How to Reach It, by J. G. Richardson, M D., of Philadelphia, Professor of Hygiene in the University of Pennsylvania, etc. III. Sea-Air and Sea-Bathing, by William S. Forbes, M.D., of Philadelphia, Surgeon to the Episcopal Hospital, etc. IV. The Summer and its Diseases, by James C. Wilson, M.D., of Philadelphia, Lecturer on Physical Diagnosis in Jefferson Medical College, etc. V. Eyesight, and How to Care for It, by George C. Harlan, M.D., of Philadelphia, Surgeon to the Wills (Eye) Hospital. VI. The Throat and the Voice, by J. Solis Cohen, M. D., of Philadelphia, Lecturer on Diseases of the Throat in Jefferson Medical College. VII. The Winter and its Dangers, by Hamilton Osgood, M.D., of Boston, Editorial Staff Boston Medical and Surgical Journal. VIII. The Mouth and the Teeth, by J. W. White, M.D., D. D.S., Editor of the Dental Cosmos. IX. Our Homes, by Henry Hartshorne, M.D., of Philadelphia, formerly Professor of Hygiene in the University of Pennsylvania. X. The Skin in Health and Disease, by L. D. Bulkley, M.D., of New York, Physician to the Skin Department of the Demilt Dispensary and of the New York Hospital. XI. Brain Work and Overwork, by H. C., Wood, jr., M.D., of Philadelphia, Clinical Professor of Nervous Diseases in the University of Pennsylvania. We have received the first two numbers and find them excellent.

*CLINICAL LECTURES ON DISEASES PECULIAR TO WOMEN*, by LOMBE ATTHILL, M.D., University of Dublin. Master of the Rotunda Hospital, Dublin; consulting Obstetric Surgeon to the Adelaide Hospital; ex-president of the Dublin Obstetrical Society, etc , etc. Fifth edition, revised and enlarged, with illustrations. Philadelphia: Lindsay & Blakiston. 1879.

The above is the fifth edition of these lectures, in which we think the author has successfully accomplished his object, "To furnish to practitioners and students, within the limits of a moderate-sized volume, such an account of the diseases peculiar to women, verified by his personal experience, as would meet their wants." The work contains 342 pages, neatly gotten up, illustrated, tersely written, interesting and eminently practical.

